# Inequalities of the Waiting Time for Education Health and Care Plan Provision for Pupils With Intellectual Developmental Disabilities: A Brief Report

**DOI:** 10.1111/jir.13239

**Published:** 2025-04-23

**Authors:** Irene O. Lee, Jeanne Wolstencroft, Harriet Housby, Marianne B. M. van den Bree, Samuel J. R. A. Chawner, Jeremy Hall, Michael J. Owen, IMAGINE ID Consortium*, David H. Skuse

**Affiliations:** ^1^ Behavioural and Brain Sciences Unit, Population Policy and Practice Programme, Great Ormond Street Institute of Child Health University College London London UK; ^2^ Centre for Neuropsychiatric Genetics and Genomics, Division of Psychological Medicine and Clinical Neurosciences Cardiff University Cardiff UK; ^3^ Neuroscience and Mental Health Innovation Institute, Division of Psychological Medicine and Clinical Neurosciences Cardiff University Cardiff UK

**Keywords:** education health and care plan, index of multiple deprivation, inequality, intellectual and developmental disabilities, regions of England, socio‐economical status, waiting time

## Abstract

**Background:**

Children and young people with intellectual and developmental disabilities (IDDs) face challenges across various aspects of their lives and require significant support, particularly in the field of education. In the United Kingdom, Education, Health and Care Plans (EHCPs) support those with special educational needs (SEN) in schools. Disparities exist throughout our national educational system with respect to how long pupils with IDDs must wait for an EHCP, but the socio‐demographic influences on those disparities are currently unknown. Delays in providing EHCP support result in negative educational, wellbeing and developmental outcomes. Using data from the National Pupil Database (NPD), we examined variabilities in waiting times for EHCP provision, and correlations with potentially influential variables such as SEN classification, family socio‐economic status, region of domicile and ethnicity.

**Methods:**

This national study recruited 2131 participants [6–28 years old, mean (SD) = 14.1 (4.4) years] with IDDs associated with a genetic condition. Families gave consent for their child's educational records to be reviewed by the research team. All participants had received an EHCP at some point during their full‐time education in England. We accessed the NPD (provided by the UK Department for Education), for details of participants' primary SEN type, free school meal eligibility, ethnicity and the academic year in which they received an EHCP. Based on their home address postcode, we assigned to each family an index of multiple deprivation (IMD) score. From the NPD, we calculated the waiting time between a child's recommendation for an EHCP and the time they received it. We compared these data with IMD scores, primary SEN type, free school meal eligibility, English region of domicile, ethnicity, and sex. We used linear regression models to examine the associations between the predictors (the above demographic independent variables) and the duration of time it took for children to receive an EHCP.

**Results:**

Participants with IDDs of genetic aetiology who lived in the most socially deprived regions of England waited longer for EHCP support than those in the least deprived regions, irrespective of the NPD classification of the child's SEN type. Neither the child's ethnicity nor their sex had any added impact. Whatever their IMD status, participants living in London obtained an EHCP more quickly than those living elsewhere in England.

**Conclusions:**

There are nationwide inconsistencies in the time taken to provide EHCPs to children and young people whose intellectual impairments are of known genetic aetiology. Regional inequalities in the funds available to local education authorities could be a major contributory factor.

**Abbreviations**
EHCP: Education Health and Care Plan
IDD: intellectual and developmental disability
IMAGINE‐ID: Intellectual Disability and Mental Health: Assessing the Genomic Impact on Neurodevelopment
IMD: Index of Multiple Deprivation
LEA: Local Education Authority
NPD: National Pupil Database
SD: standard deviation
SEN: special educational needs
χ2: chi‐squared

## 1 Background

Children and young people with intellectual and developmental disabilities (IDDs) comprise 1.7% of the population worldwide and are impacted in all key areas of life, including health, education and wellbeing (Nair et al. 2022). IDD is a heterogeneous group of disorders characterised by significantly impaired intellectual functioning and deficits in adaptive behaviours (Bertelli et al. 2016; Ilyas et al. 2020). IDDs of genetic aetiology are associated with a wide range of other disorders, such as autism, anxiety and behavioural problems (Wolstencroft et al. 2022). Most have complex educational needs, compared to typical developing pupils. In the United Kingdom, state‐funded schools with IDD students who have special educational needs (SEN) can apply for Education, Health and Care Plans (EHCP), which provide financial aid to support those pupils' specific needs. Government guidelines recommend a maximum of 20 weeks for the local education authority (LEA) to assess a pupil in need and produce a final EHCP recommendation (UK Government 2024a). It is recognised that EHCPs are not provided to all pupils with substantial educational needs (Richards 2022), and many are declined an EHCP after the first application is made. Whether the recommendation is successful or not depends on the response of the LEA, which finances the provision. If the assessment recommends an EHCP, the child will receive support. However, if the assessment does not recommend an EHCP, the parents or the young persons have the right to appeal to the Special Educational Needs and Disability Tribunal (UK Government 2024b). The duration of time between the original request for an assessment and the EHCP recommendation varies substantially, depending on the region of England in which they live (Marsh and Howatson 2020). It can be far longer than 20 weeks for two main reasons: first, because the original assessment had not been completed within that 20‐week period and second, because the proportion of assessments that lead to a recommendation, which is acceptable to the child's parents is highly variable (Atkinson et al. 2024). The longer the period between the initial assessment and the eventual provision of special educational support, the greater the risk to the child's educational, health and developmental progress (Emerson 2012; Lőrinc et al. 2020; Parker et al. 2016).

In the present study, we investigated the influence of several key variables that we hypothesised could affect the duration of time between a recommendation for assessment and the provision of an EHCP (Lee et al. 2024). All children and young people in this study were eligible for SEN support. We assessed the impact of the following key variables on waiting times between the first request for support and the granting of an EHCP: the child's primary SEN type, the family's socio‐economic status [index of multiple deprivation (IMD) and free school meal eligibility], English region of domicile, ethnicity and sex.

## 2 Methods

### 2.1 Study Participants

2131 participants with IDDs were recruited at ages from 6 to 28 years [mean (SD) = 14.1 (4.4) years; 57% male, see Table 1]. All had pathogenic genetic conditions, as reported by UK regional genetics centres or other private clinics, as part of a previous IMAGINE‐ID study (Chawner et al. 2019; Lee et al. 2024; Wolstencroft et al. 2022). All participants had studied in state schools, where a LEA provided funding. We obtained comprehensive educational data on participants (from ages 4 to 19) from the National Pupil Database for England (NPD). All participants had (eventually) been granted an educational health and care plan (EHCP) by their LEA (Lee et al. 2024). The London Square Research Ethics Committee provided ethical approval for this study. Parents/caregivers of children under the age of 16 (or participants over 16 years of age) gave written or online consent.

**TABLE 1:** Participant demographic information.

Demographic category	Frequency, *N* (% of total count)	95% CI cohort proportion
Total	2131	
Sex		
Male	1208 (56.7%)	54.6%–58.8%
Female	923 (43.3%)	41.2%–45.4%
Age at recruitment, years		
Mean (SD) [95% CI]	14.1 (4.4)	[13.9–14.3]
Median	13.5	
Range	6.4–27.7	
Age at being identified as SEN pupil		
Mean (SD) [95% CI]	5.3 (1.4)	[5.2–5.3]
Median	5.0	
Ethnicity		
White	1885 (88.5%)	87.1%–89.9%
Asian	104 (4.9%)	4.7%–5.8%
Black	21 (1.0%)	0.9%–1.0%
Mixed	110 (5.2%)	4.3%–6.1%
Any other ethnic group	11 (0.5%)	0.4%–0.9%
Primary SEN type		
Profound and multiple learning difficulty	221 (10.4%)	6.4%–14.4%
Severe learning difficulty	619 (29%)	25.4%–32.5%
Moderate learning difficulty	322 (15.1%)	11.2%–19.0%
Specific learning difficulty	129 (6.1%)	2.0%–10.2%
Speech language communication needs	338 (15.9%)	13.9%–17.9%
Autism spectrum disability	367 (17.2%)	13.3%–21.1%
SEMH + BESD	46 (2.2%)	2.0%–6.4%
MSI + hearing + visual impairment	16 (0.8%)	0.7%–0.9%
Physical disability	43 (2.0%)	1.8%–2.2%
Other difficulties/disabilities	30 (1.4%)	0.1%–2.7%
Index of multiple deprivation decile		
1 (most deprived)	191 (9.0%)	7.8%–10.2%
2	204 (9.6%)	8.3%–10.9%
3	209 (9.8%)	8.5%–11.1%
4	187 (8.8%)	7.6%–10.0%
5	209 (9.8%)	8.5%–11.1%
6	217 (10.2%)	8.9%–11.5%
7	204 (9.6%)	8.3%–10.9%
8	223 (10.5%)	9.2%–11.8%
9	237 (11.1%)	9.8%–12.4%
10 (least deprived)	253 (11.8%)	10.4%–13.2%
Free school meal eligibility		
No	1425 (66.9%)	64.5%–69.3%
Yes	706 (33.1%)	29.6%–36.6%
Regions of England		
North East	88 (4.1%)	3.3%–4.9%
North West	182 (8.5%)	7.3%–9.7%
Yorkshire/Humber	277 (13.0%)	11.6%–14.4%
East Midlands	132 (6.2%)	5.2%–7.2%
West Midlands	296 (13.9%)	12.4%–15.4%
East England	336 (15.8%)	14.3%–17.3%
South East	413 (19.4%)	17.7%–21.1%
South West	88 (4.1%)	3.3%–4.9%
London	233 (10.3%)	9.0%–11.6%

Abbreviations: BESD = Behavioural Emotional Social Difficulty, CI = confidence interval, MSI = Multi‐Sensory Impairment, *N* = number of cases, SD = standard deviation, SEMH = Social, Emotional and Mental Health, SEN = special educational need.

### 2.2 Data Source and Description

We obtained educational histories from the NPD. The UK Department for Education manages this resource. The NPD (England) educational dataset was ideal for investigating influences on EHCP waiting times, because all participants were eligible for special educational support and attended English mainstream or special education state‐funded schools, which record information about EHCP provisions. We therefore had access to official records, ensuring accuracy. Data were available for the period of 2006–2021. Educational information included the primary SEN type, ethnicity, socio‐economic impoverishment (whether the child was eligible for free school meal), the year the pupil was first identified as having SEN and the year that pupil was granted an EHCP. A variable, ‘EHCP waiting time’, was estimated by taking the difference in time between the year the pupil's need for assessment was recognised and the year they received the EHCP. Exact dates were not available in the NPD datasets, so we computed the variable ‘Age at first record of obtaining an EHCP’ by subtracting the child's year of birth from the year in which they first received an EHCP.

There are several potential reasons why an EHCP could be granted, and these were recorded as ‘SEN type’. Records often showed that individual pupils were classified as belonging to different ‘primary SEN type’ in consecutive academic years. For the purpose of this analysis, we assigned just one primary SEN type for each pupil, which was the one most frequently recorded in their education history (Lee et al. 2024). Knowing the postcode for the child's family home allowed us to assign an IMD. Although IMD scores are continuous, they are often presented in deciles (1 = *most deprived*, 10 = *least deprived*) (UK Government 2019, 2021). Home addresses were categorised into nine regions of England according to the UK Office for National Statistics (UK Government 2024c).

### 2.3 Statistical Analysis

We used parametric statistics to identify the impact of independent variables including IMD decile, primary SEN type, region of England, free school meal eligibility, ethnicity and sex on EHCP waiting times (the dependent variable). Bonferroni corrections were applied to multiple comparisons. Chi‐squared (*X* ^2^) tests compared categorical variables. We used stepwise linear regression models to identify the most salient impactful variables on EHCP waiting time using the aforementioned demographic variables as covariates. A *p* value < 0.05 was adopted in all the analyses of this study as a cut‐off of statistical significance. All data analyses were performed in SPSS Version 28 on the Office for National Statistics Secure Research Service platform. Following the statistical disclosure guidelines from the UK Office for National Statistics, counts of fewer than ten pupils in subcategories of the NPD cannot be published because of potential identification. Therefore, exact numbers for certain subgroups cannot be reported in this article.

## 3 Results

The average age at which a pupil's record indicated they had been granted an EHCP was 7.1 years of age (SD = 2.6) [mean (standard deviation)]. There were no differences in that mean age between boys and girls, *t*(2129) = −0.49, *p* = 0.31 (see Table 2). The average EHCP waiting time was 1.68 (2.2) years. There was no difference between mean wait times for boys and girls, *t*(2129) = 0.13, *p* = 0.90, and we observed no differences between ethnic groups, *F*(4, 2126) = 0.55, *p* = 0.70.

**TABLE 2:** Comparisons of the age of first record of obtaining an education health and care plan (EHCP) and the waiting time for an EHCP by sex, index of multiple deprivation (IMD) decile, free school meal eligibility, primary SEN type, region of domicile and ethnicity.

Variable	EHCP waiting time, year		*t* or *F*	Effect size	*p*
	Mean (SD) [*N*]	95% CI		*d* or *ŋ* ^*2*^	
Age of first record of obtaining an EHCP, years old			−0.49	0	0.31
Male	7.1 (2.6) [1208]	7.0–7.2			
Female	7.1 (2.7) [923]	7.0–7.4			
Total	7.1 (2.6) [2131]	7.1–7.3			
Sex			0.13	0.01	0.90
Male	1.68 (2.2) [1208]	1.51–1.88			
Female	1.67 (2.2) [923]	1.52–1.82			
Total	1.68 (2.2) [2131]	1.58–1.77			
Ethnicity			0.55	0.001	0.70
White	1.69 (2.2) [1885]	1.59–1.78			
Asian	1.44 (2.0) [104]	1.05–1.84			
Black	1.48 (1.7) [21]	0.69–2.26			
Mixed	1.84 (2.3) [110]	1.39–2.28			
Any other ethnic group	1.36 (1.4) [11]	0.45–2.28			
IMD decile			7.05	0.03	*< 0.001*
1 (most deprived)	2.45 (2.5) [191]	2.09–2.8			
2	2.11 (2.4) [204]	1.79–2.44			
3	1.91 (2.2) [209]	1.61–2.22			
4	1.88 (2.3) [184]	1.53–2.22			
5	1.71 (2.2) [209]	1.41–2.02			
6	1.41 (2.0) [217]	1.15–1.68			
7	1.3 (1.9) [204]	1.03–1.88			
8	1.59 (2.2) [223]	1.30–1.88			
9	1.52 (2.0) [237]	1.26–1.78			
10 (least deprived)	1.14 (1.8) [253]	0.91–1.36			
Free school meal eligibility			−6.06	0.28	*< 0.001*
No	1.47 (2.1) [1425]	1.36–1.58			
Yes	2.10 (2.4) [706]	1.93–2.27			
Primary SEN type			33.74	0.13	*< 0.001*
Profound and Multiple LD	0.62 (1.3) [221]	0.44–0.79			
Severe LD	0.92 (1.5) [619]	0.8–1.03			
Moderate LD	2.46 (2.5) [322]	2.19–2.73			
Specific LD	3.02 (2.6) [129]	3.02–3.48			
Speech language communication needs	1.96 (1.2) [338]	1.73–2.19			
Autism spectrum disorder	2.18 (2.5) [367]	1.92–2.44			
SEMH + BESD	2.93 (2.2) [46]	2.28–3.59			
MSI + Hearing + visual impairment	0.88 (2.1) [16]	0.1–1.97			
Physical disability	0.74 (1.3) [43]	0.34–1.15			
Other difficulties/disabilities	1.53 (1.9) [30]	0.83–2.23			
Region of domicile			3.83	0.01	*< 0.001*
North East	2.27 (2.8) [88]	1.68–2.87			
Yorkshire/Humber	1.96 (2.4) [277]	1.68–2.25			
East Midlands	1.90 (2.3) [132]	1.50–2.30			
East England	1.83 (2.3) [336]	1.59–2.08			
West Midlands	1.72 (2.1) [296]	1.47–1.97			
North West	1.69 (2.1) [182]	1.39–2.0			
South East	1.52 (2.1) [413]	1.31–1.72			
South West	1.32 (2.0) [174]	1.02–1.61			
London	1.25 (1.6) [233]	1.04–1.46			

Abbreviations: BESD = Behavioural Emotional Social Difficulty, CI = confidence interval, *d* = Cohen's *d*, LD = learning difficulty, MSI = Multi‐Sensory Impairment, *N* = number of cases, ŋ^2^ = eta squared, SD = standard deviation, SEMH = Social, Emotional and Mental Health, SEN = special educational Need.

### 3.1 Socio‐Economic Status: IMD Decile and Free School Meal Eligibility

**FIGURE 1:**
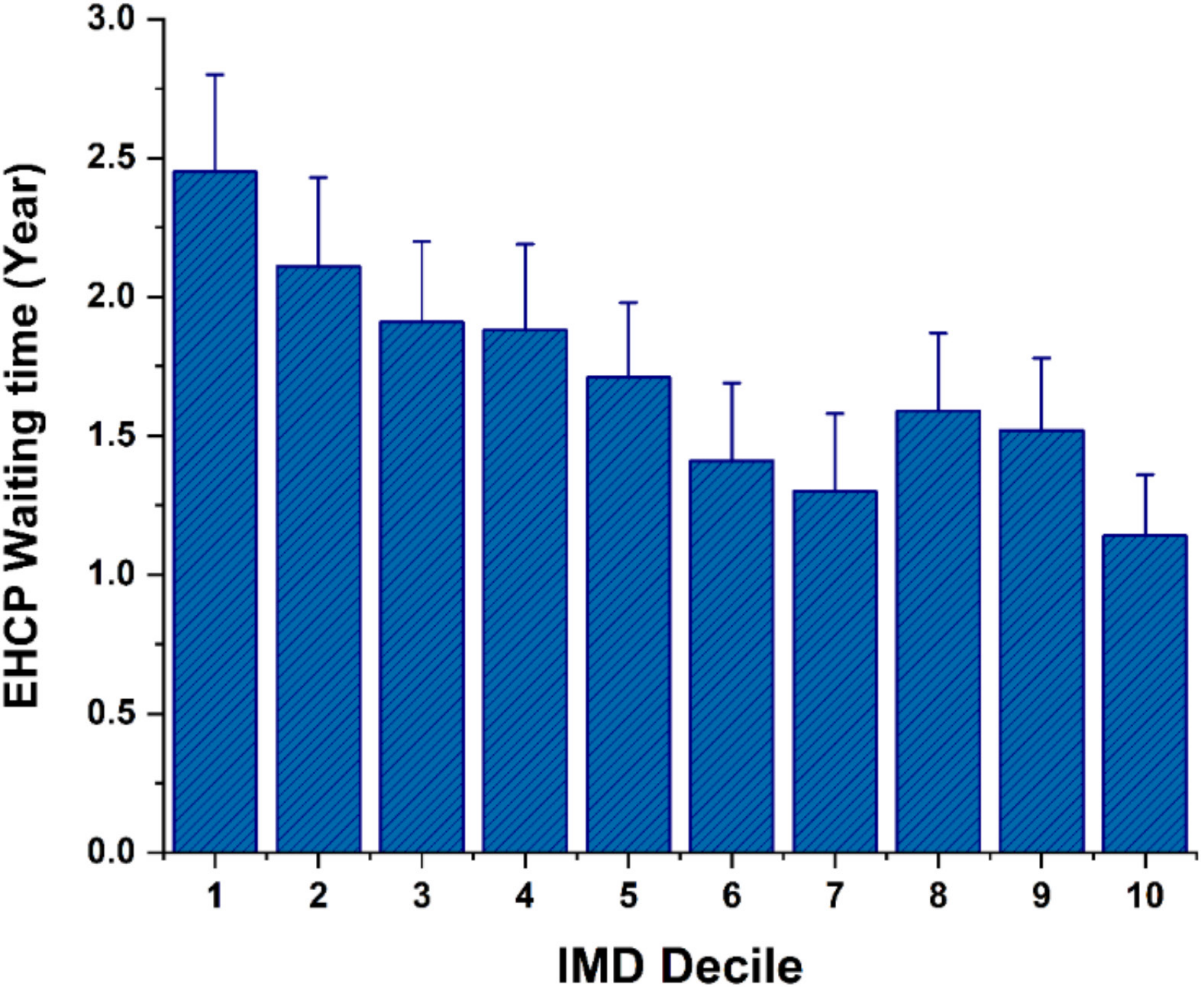
The waiting times for an education health and care plan (EHCP) of the cohort participants living in the ten index of multiple deprivation (IMD) deciles in England. Mean and 95% CI error bars are shown. IMD decile 1 = *most deprived area*, decile 10 = *least deprived areas*.

The participants who lived in the more deprived areas (lower IMD deciles) had waited significantly longer for an EHCP than those in the least deprived areas (higher IMD deciles), *F*(9, 2121) = 7.05, *p* < 0.001 (see Table 2 and Figure 1). The waiting time of participants living in IMD decile 10 (least deprived areas) was 1.14 (1.8) years (95% CI = 0.91–1.36). This was significantly shorter than that of those living in the most deprived IMD decile 1 (*p* < 0.001), IMD decile 2 (*p* < 0.001), IMD decile 3 (*p* = 0.006) and IMD decile 4 (*p* = 0.02) by multiple comparison tests. Children living in IMD decile 1 (most deprived areas) [2.45 (2.5) years, 95% CI = 2.09–2.80] had the longest EHCP waiting time, significantly longer than for those living in IMD deciles from 5 to 10 (*p* values between 0.032 and < 0.001). Overall, EHCP waiting times were associated with IMD deciles, with shorter waiting time for pupils from the least deprived areas.

Participants who were eligible for free school meals had a waiting time of 2.03 (2.3) years, which was significantly longer than for those who were not eligible [1.42 (2.0) years], *t*(2129) = −6.06, *p* < 0.001 (see Table 2), and they were more likely to live in the most deprived areas, *X* ^2^(9) = 260.50, *p* < 0.001.

### 3.2 Primary SEN Type

Primary SEN subtypes are listed in Table 2. There were statistically significant differences in waiting time between SEN subtypes, *F*(9, 2121) = 33.74, *p* < 0.001. Participants with profound and multiple learning difficulties had the shortest waiting time [0.62 (1.3) years (95% CI = 0.44–0.79)]. Pupils with specific learning difficulties waited the longest time [3.02 (2.6) years, 95% CI = 2.57–3.48].

**FIGURE 2:**
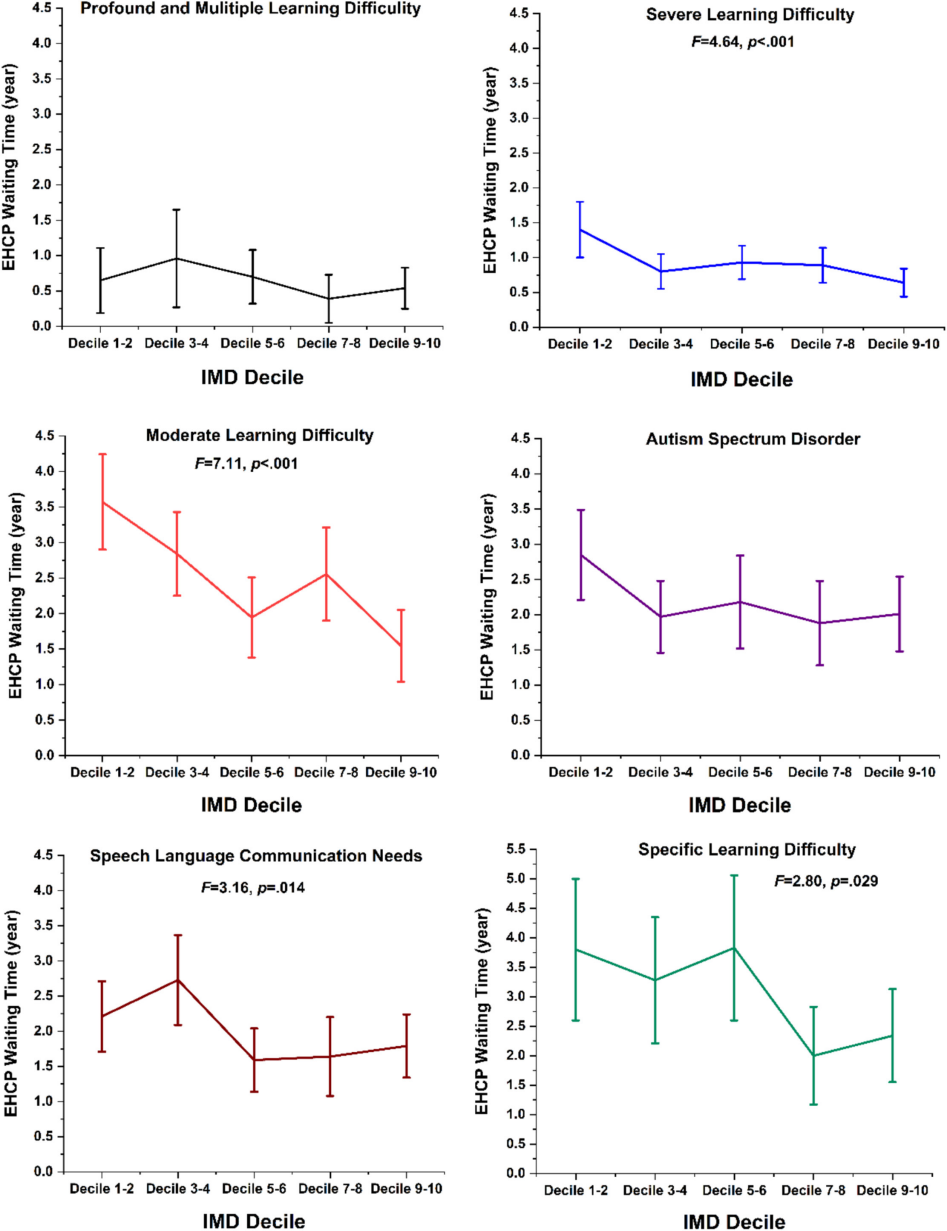
The education health and care plan (EHCP) waiting time of each primary special education need type in different index of multiple deprivation (IMD) quintiles, with 95% confidence interval error bars. IMD decile 1–2 = *most deprived areas*, decile 9–10 = *least deprived areas*.

We investigated differences in waiting times for an EHCP in different IMD quintiles within the same primary SEN type. Overall, participants living the more deprived areas waited significantly longer than those in the least deprived areas (Figure 2) irrespective of their SEN type. Even within each SEN subtype, waiting times varied significantly by IMD quintile: severe learning difficulty *F*(4, 2126) = 4.64, *p* < 0.001, ŋ^2^ = 0.029; moderate learning difficulty, *F*(4, 2126) = 7.11, *p* < 0.001, ŋ^2^ = 0.082; speech language communication needs, *F*(4, 2126) = 3.16, *p* = 0.014, ŋ^2^ = 0.037 and specific learning difficulty, *F*(4, 2126) = 2.80, *p* = 0.029, ŋ^2^ = 0.083.

### 3.3 Region of Domicile

**FIGURE 3:**
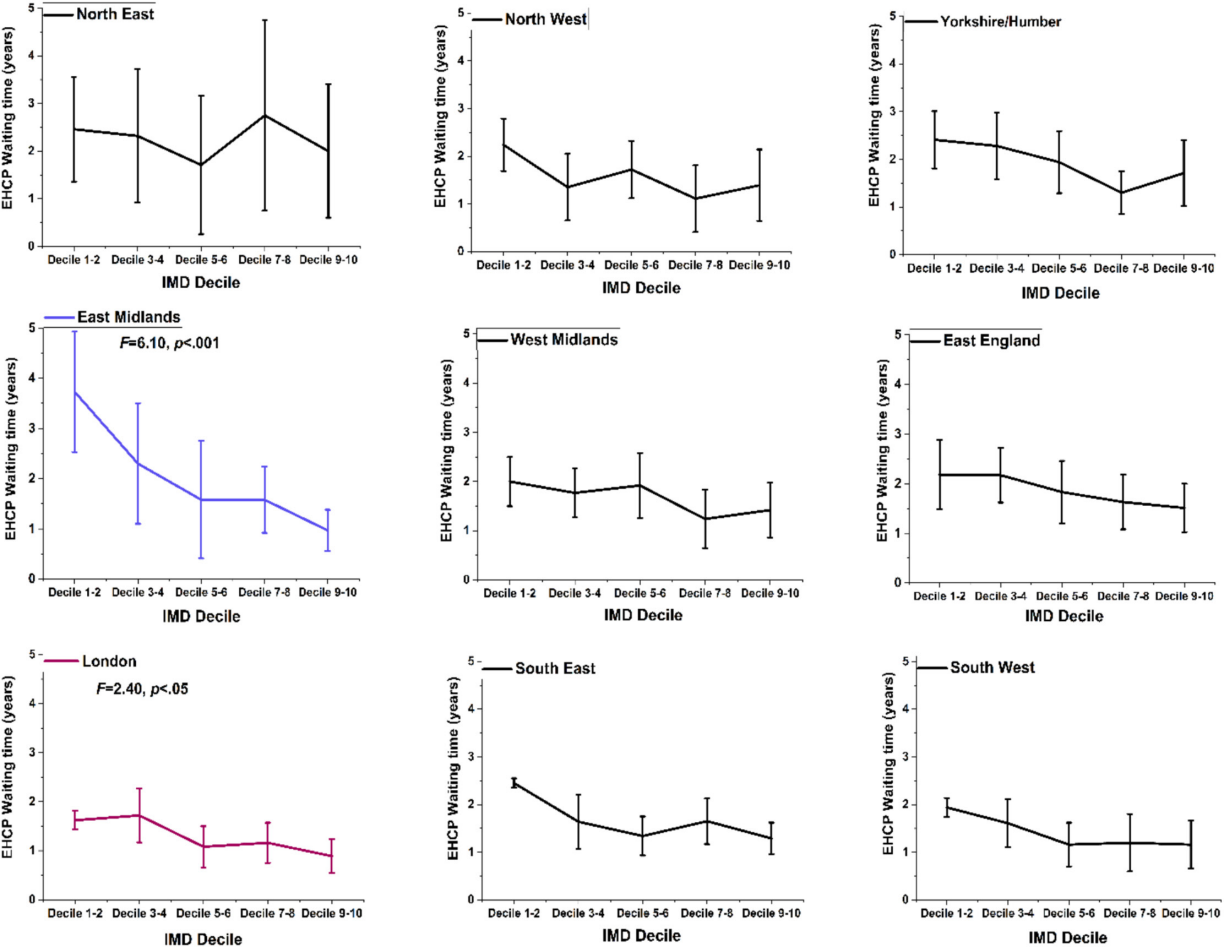
The education health and care plan (EHCP) waiting time at different index of multiple deprivation (IMD) quintiles in each region of England, with 95% confidence interval error bars. IMD decile 1–2 = *most deprived areas*, decile 9–10 = *least deprived areas*.

Statistical analysis revealed differences between EHCP waiting times for different regions of England, *F* = 3.83, *p* < 0.001 (see Table 2). Pupils from London had the shortest EHCP waiting time [1.25 (1.6) years, 95% CI = 1.04–1.46] whereas those in the North East had the longest time [2.27 (2.8) years, 95% CI = 1.68–2.87], compared to the other domiciles. Bonferroni corrected pairwise comparisons showed significant differences between London and Yorkshire and the Humber (*p* = 0.006), as well as between London and West of England (*p* = 0.002). The variables IMD decile and region of domicile were significantly correlated, *r*(2129) = 0.20, *p* < 0.001. We investigated the waiting time for an EHCP in each region of domicile in England and measured this in comparison to IMD quintiles (see Figure 3). Within two regions, that is, East Midlands, *F*(4, 2126) = 6.10, *p* < 0.001, ŋ^2^ = 0.16 and London, *F*(4, 2126) = 2.40, *p* < 0.05, ŋ^2^ = 0.04, pupils living in areas of greatest deprivation waited significantly longer than those living in relatively advantaged areas. London has the shortest waiting times for children living in all IMD quintiles compared to those in all the other regions of England. There were also differences in the proportion of participants eligible for free school meals amongst the nine regions in England *X* ^2^(8) = 29.86, *p* < 0.001 (see Table 3). London (32%) and North East England (43%) had the second lowest and the highest proportion of pupils with free school meal eligibility, respectively. In general, the higher the proportion of children living in an English region who were eligible for free school meals (i.e., had low family incomes), the longer the EHCP waiting times were within that region (see region of domicile in Tables 1 and 3).

**TABLE 3:** The free school meals (FSM) eligibility of the participants living in the nine regions of England.

FSM eligibility	No	Yes	*X* ^2^	*p*
Region of domicile			29.86	< 0.001
North East	50 (56.8)	38 (43.2%)		
Yorkshire/Humber	162 (58.5%)	115 (41.5%)		
East Midlands	82 (62.1%)	50 (37.9%)		
West Midlands	191 (64.5%)	105 (35.5%)		
North West	119 (65.4%)	63 (34.6%)		
South West	116 (66.7%)	58 (33.3%)		
East England	231 (68.8%)	105 (31.3%)		
London	163 (70%)	70 (30%)		
South East	311 (75.3%)	102 (24.7%)		

Stepwise linear regression models estimated the influence of a range of demographic variables on EHCP waiting time. Sex and ethnicity were not significant predictors. In contrast, IMD decile and free school meal eligibility remained strong predictors when controlling for sex, ethnicity and region of domicile in England (Table 4). Several English regions were still associated with longer waiting times, even when IMD decile and free school meal eligibility had been controlled within a stepwise analysis.

**TABLE 4:** Stepwise linear regression models evaluating, stepwise, the impact of demographic variables on the duration of waiting times for provision of an education health and care plan.

Model	Independent variable(s)	B (SE)	Standardised β	*p*
1	R‐squared = 2.7%			
	Constant	2.377 (0.113)		*< 0.001*
	IMD decile	−0.121 (0.016)	−0.161	*< 0.001*
	Ethnicity (White as ref)			
	Asian	−0.388 (0.22)	−0.038	0.076
	Black	−0.524 (0.48)	−0.024	0.272
	Mixed	0.117 (0.21)	0.012	0.581
	Others	−0.702 (0.58)	−0.026	0.226
	Sex (Male as ref)	0.038 (0.10)	0.009	0.688
2	R‐square = 3.6%			
	Constant	1.973 (0.185)		*< 0.001*
	IMD decile	−0.114 (0.017)	−0.152	*< 0.001*
	Ethnicity (White as ref)			
	Asian	−0.319 (0.221)	−0.031	0.149
	Black	−0.341 (0.48)	−0.015	0.478
	Mixed	0.159 (0.213)	0.016	0.456
	Others	−0.574 (0.58)	−0.021	0.323
	Sex (Male as ref)	0.042 (0.095)	0.010	0.657
	Region of England (London as ref)			
	North East	0.839 (0.27)	0.076	*0.002*
	Yorkshire/the Humber	0.561 (0.195)	0.086	*0.004*
	East Midlands	0.609 (0.236)	0.067	*0.010*
	North West	0.246 (0.217)	0.031	0.258
	West Midlands	0.326 (0.191)	0.052	0.089
	East England	0.519 (0.187)	0.086	*0.005*
	South East	0.309 (0.179)	0.056	0.085
	South West	0.029 (0.219)	0.004	0.896
3	R‐squared = 4.4%			
	Constant	1.699 (0.197)		*< 0.001*
	IMD decile	−0.092 (0.018)	−0.122	*< 0.001*
	Ethnicity (White as ref)			
	Asian	−0.249 (0.221)	−0.025	0.259
	Black	−0.378 (0.479)	−0.017	0.430
	Mixed	0.108 (0.212)	0.011	0.610
	Others	−0.586 (0.578)	−0.022	0.311
	Sex (Male as ref)	0.063 (0.095)	0.014	0.504
	Region of England (London as ref)			
	North East	0.813 (0.272)	0.074	*0.003*
	Yorkshire/the Humber	0.537 (0.195)	0.083	*0.006*
	East Midlands	0.581 (0.235)	0.064	*0.014*
	North West	0.259 (0.216)	0.033	0.231
	West Midlands	0326 (0.191)	0.051	0.088
	East England	0.520 (0.186)	0.087	*0.005*
	South East	0.318 (0.178)	0.057	0.075
	South West	0.015 (0.218)	0.002	0.944
	Free school meal eligibility			
	Yes (No as ref)^a^	0.42 (0.106)	0.09	*< 0.001*

Abbreviations: IMD = index of multiple deprivation, ref = reference, SE = standard error.

^a^ Free school meal eligibility was significantly correlated to IMD decile and sex (all *p* values < 0.001); however, addition of this variable in the stepwise regression did not affect the outcome.

## 4 Discussion

This study aimed, for the first time, to find potential influences on the waiting time for obtaining an EHCP, for children and young people with intellectual and developmental disabilities of genetic aetiology. We found evidence for the impact of socio‐economic status (including IMD decile and free school meal eligibility), primary SEN type and other demographic factors including domicile in different English regions. We did not find any influence of ethnicity or sex. We show that families who lived in areas with greatest socio‐economic deprivation, both in terms of neighbourhood and in terms of English region, waited significantly longer for an EHCP irrespective of the severity of the child's learning difficulties. In general, children with more impairing and observable disabilities, such as profound and multiple learning difficulties, physical disabilities and sensory impairments (visual or hearing impairments), had the shortest waiting times. Children whose SEN type was primarily behavioural or emotional with social difficulties waited longer.

In this cohort, participants who live in conditions of greatest socio‐economic deprivation, many of whom have children who were eligible for free school meals, waited the longest, irrespective of the English region. We already know that socio‐economically disadvantaged children do not receive the support they need to succeed in education and other social environments (Anders and Henderson 2019). Lack of support perpetuates existing inequalities. It seems plausible that parents from more socio‐economically advantaged backgrounds, with a higher income and better educational background, who have a wider network to ask for advice, will press their LEA harder to provide support for their child. Inequalities in socio‐economic status affected waiting times, irrespective of the child's degree of disability. In theory, the timescale for provision of SEN support in schools should be the same for all children with the same SEN type irrespective of where they live. In general, those with less severe problems, such as specific learning difficulties or emotional and behavioural disorders had to wait 3–4 years wherever they lived. Delays in providing support tend to result in multiple negative outcomes, including disengagement, exclusion, poor academic and health progress together with long‐term physical and mental health problems (Emerson 2012; Lőrinc et al. 2020; Parker et al. 2016). Some of these negative pressures are more subtle and not captured by an SEN classification. For instance, pupils with behavioural and social deficits are more likely to be bullied (Davis 2012; Göransson and Bengtsson 2023; Pedace 2009). Delays in supplying an EHCP may worsen their mental wellbeing.

The regional differences we found in waiting times show there are disparities in regional resources for providing support to the cohort. Participants living in London from all IMD quintiles had notably shorter waiting times compared to those living in other, less economically advantaged regions of England. Inequalities in provisions based on the degree of familial social deprivation were particularly severe in the East Midlands (Figure 3). There was a clear association between longer EHCP waiting times and regions of the country that had a higher proportion of participants receiving free school meals, implying generalised socio‐economic deprivation. This echoes a recent report that regional variations in the gap between the educational progress of advantaged and persistent disadvantaged children in the general population have persisted over the past decade (Hutchinson et al. 2020).

Many SEN pupils will not be granted an EHCP following their first application. Parents, schools or the young persons can appeal to their local education authority if they do not agree with the decision (Cullen and Cullen 2021; UK Government 2024b). An appeal process increases the waiting time until a pupil is granted an EHCP (Marsh and Howatson 2020). Navigating the appeals procedure favours parents who live in more advantaged circumstances, who have had a better education and who have access to an extensive social network to help with advice.

Wide variations exist between education authorities in English regions in the proportion of pupils granted an EHCP (5.2%–1.0%) (Marsh and Howatson 2020). One factor that influences regional disparities is the success of tribunal appeals (Marsh and Howatson 2020). This in turn could be attributed to regional variations in financial resources. Decisions about which regions get the greatest financial support for education are based on a historic national funding formula, which the House of Commons Education Committee suggested in 2019 should be changed (House of Commons 2019; Marsh and Howatson 2020; National Audit Office 2019).

There are some limitations to this study. First, we can only approximate the variable EHCP waiting time in years, because it was calculated as the difference between the year the pupil was first identified as having SEN and the year in which they were granted an EHCP. No exact dates or months are provided by the NPD datasets. The second limitation relates to the necessity of selecting a single primary special educational need type to represent a single pupil. We observed that, in practice, pupils were classified within multiple primary special education need types during their education in different academic years. We recognise that we have identified relatively weak predictors of waiting times. In our regression models, the only a small proportion of the variance in that dependent variable was explained by the independent variables. We cannot generalise to other conditions that are common reasons for EHCP requests, such as ADHD. All children who participated in the IMAGINE‐ID study had intellectual disabilities associated with pathogenic genetic variants and would often have physical phenotypes as well as behavioural and intellectual difficulties, therefore may have attracted more attention within mainstream education at an early stage.

## 5 Conclusions

This study is the first to present a systematic analysis of the influences of socio‐economic inequalities on waiting times for an EHCP for children and young people with intellectual and developmental disabilities of genetic origin. Our national cohort study revealed disparities that indicated those wait‐times were predominantly influenced by socio‐economic factors rather than by the severity or type of a child's disability. Families living in conditions of greatest deprivation waited over twice as long as families from advantaged backgrounds, irrespective of the region of the country in which their child was educated. The study has implications for policy, emphasising that LEA need to monitor responses to requests for an EHCP and ensure there is equity of provision for all children with disabilities, irrespective of their socio‐economic background, to remove barriers for families from low socio‐economic categories accessing EHCP support; and have a unified national approach to eliminate geographical inconsistencies. It also implies that following a neurodevelopmental genetic diagnosis, a referral pathway for educational support would lessen the burden on families.

## Ethics Statement

The study was approved by the London Square Research Ethics Committee in the UK (14/LO/1069). Written or online consent was obtained from the parents/caregivers of children under the age of 16 or from the consultees or the participants over aged 16 who took part in this study.

## Conflicts of Interest

The authors declare no conflicts of interest.

## Data Availability

The data from the Department for Education (DfE), UK, that support the findings in this paper are not available for data sharing according to the guidelines of the DfE and the Office for National Statistics (ONS). Other research data from this study are available from the authors upon application. This work contains statistical data from ONS, which is Crown Copyright. The use of the ONS statistical data in this work does not imply the endorsement of the ONS in relation to the interpretation or analysis of the statistical data. This work uses research datasets that may not exactly reproduce National Statistics aggregates.

## IMAGINE ID Consortium Members

**Table:**

Surname	Initials	First name	Title	Institution
Housby	H	Harriet	Ms	Great Ormond Street Institute of Child Health, University College London, UK
Lee	I	Irene	Mrs	Great Ormond Street Institute of Child Health, University College London, UK
Skuse	D	David	Professor	Great Ormond Street Institute of Child Health, University College London, UK
Wolstencroft	J	Jeanne	Dr	Great Ormond Street Institute of Child Health, University College London, UK
Mandy	W	William	Dr	Division of Psychology & Language Sciences, University College London, UK
Denaxas	S	Spiros	Dr	Institute of Health Informatics, University College London, London, UK
van den Bree	MBM	Marianne	Professor	Centre for Neuropsychiatric Genetics and Genomics, Division of Psychological Medicine and Clinical Neurosciences, Cardiff University, UK
Chawner	SJRA	Samuel	Dr	Centre for Neuropsychiatric Genetics and Genomics, Division of Psychological Medicine and Clinical Neurosciences, Cardiff University, UK
Hall	J	Jeremy	Professor	Centre for Neuropsychiatric Genetics and Genomics, Division of Psychological Medicine and Clinical Neurosciences, Cardiff University, UK
Holmans	P	Peter	Professor	Centre for Neuropsychiatric Genetics and Genomics, Division of Psychological Medicine and Clinical Neurosciences, Cardiff University, UK
Hope‐Bell	J	Josh	Dr	Centre for Neuropsychiatric Genetics and Genomics, Division of Psychological Medicine and Clinical Neurosciences, Cardiff University, UK
Le Roux	D	Danielle	Ms	Centre for Neuropsychiatric Genetics and Genomics, Division of Psychological Medicine and Clinical Neurosciences, Cardiff University, UK
Morrin	S	Sally	Ms	Centre for Neuropsychiatric Genetics and Genomics, Division of Psychological Medicine and Clinical Neurosciences, Cardiff University, UK
Owen	MJ	Michael	Professor Sir	Centre for Neuropsychiatric Genetics and Genomics, Division of Psychological Medicine and Clinical Neurosciences, Cardiff University, UK
Sivakumar	S	Shreeya	Ms	Centre for Neuropsychiatric Genetics and Genomics, Division of Psychological Medicine and Clinical Neurosciences, Cardiff University, UK
Baker	K	Kate	Dr	Department of Medical Genetics, University of Cambridge, UK
Raymond	FLF	Lucy	Professor	Department of Medical Genetics, University of Cambridge, UK
